# Histone deacetylases as targets for antitrypanosomal drugs

**DOI:** 10.4155/fsoa-2018-0037

**Published:** 2018-07-27

**Authors:** Aline A Zuma, Wanderley de Souza

**Affiliations:** 1 Laboratório de Ultraestrutura Celular Hertha Meyer, Instituto de Biofísica Carlos Chagas Filho, Federal University of Rio de Janeiro, Rio de Janeiro, RJ, Brazil

**Keywords:** chemotherapy, histone, histone deacetylases, histone deacetylases inhibitors, post-translational modifications, *Trypanosoma cruzi*

## Abstract

Parasitic protozoa comprise several species that are causative agents of important diseases. These diseases are distributed throughout the world and include leishmaniasis, Chagas disease and sleeping sickness, malaria and toxoplasmosis. Treatment is based on drugs that were developed many years ago, which have side effects and produce resistant parasites. One approach for the development of new drugs is the identification of new molecular targets. We summarize the data on histone deacetylases, a class of enzymes that act on histones, which are closely associated with DNA and its regulation. These enzymes may constitute new targets for the development of antiparasitic protozoa drugs. Although several protozoan species are mentioned, members of the Trypanosomatidae family are the main focus of this short review.

## Lay abstract

Parasitic protozoa comprise species that are causative agents of important diseases distributed throughout the world. The available drugs for treatment were developed many years ago, might cause side effects and produce resistant parasites. The identification of new molecular targets is required for the development of new drugs. Histone deacetylases act on histones, are closely associated with DNA and thus may constitute new targets for antiparasitic therapy, especially that against trypanosomatid protozoa.

The compartmentalization of DNA in a cell nucleus due to the presence of a nuclear membrane is an important achievement in cell evolution. It plays a fundamental role in harboring DNA, which stores the information necessary to control the various cellular processes. Due to its large size, DNA remains in an extremely compacted state, which is maintained through the interaction between DNA and proteins [1–3].

The nucleus of parasitic protozoa has some special morphological features. In most of them, the nuclear membrane remains intact during mitotic division, even though the assembly of intranuclear microtubules takes place. In the case of trypanosomatids, the nucleus is smaller, and chromatin condensation does not occur during mitosis, and typical chromosomes are not observed [4]. The nucleolus is where ribosome biogenesis occurs and is located at the center of the nucleus. It displays well-defined domains such as inner and central fibrillar zones, as well as a granular zone toward the periphery of the nucleus. The condensed heterochromatin is associated with the nuclear envelope in the interphase, whereas during mitosis, the chromatin and nucleolus are more dispersed [5]. At the end of mitosis, the nucleus divides, beginning with constriction in the middle followed by organization of the nucleolus and condensation of the chromatin [4], as shown in Figure 1.

**Figure 1.**
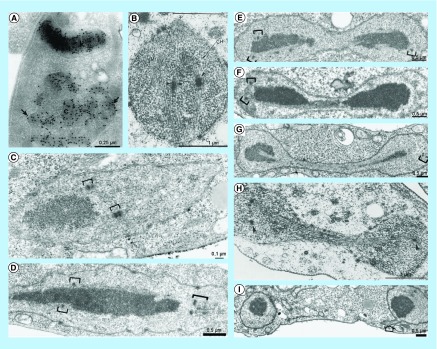
**Transmission electron microscopy of the distribution of DNA and nuclear morphology during mitosis in trypanosomatids.** **(A)** Immunodetection of DNA in *Strigomonas culicis*. During mitosis, the chromatin and also the nucleolus are more dispersed. The arrow indicated the chromatin associated with the envelope. Reproduced with permission from [5]. **(B)** The mitotic spindle is seen throughout *T. cruzi* nucleus. Reproduced with permission from [6]. **(C – G)** Different stages of mitosis in *Trypanosoma brucei*, showing the chromatin extending through the nucleus, the initial formation of central constriction and the chromatin localization at the opposite poles. Kinetochores are represented by open brackets. Reproduced with permission from *Chromosoma* (2000) [7]. **(H)** The mitotic spindle present during the central constriction of the nucleus. Reproduced with permission from *Chromosoma* (1980) [6]. **(I)** Late mitosis when the nucleus is divided into two. The final connection between the nuclei can be observed (arrow). Reproduced with permission from *Chromosoma* (2000) [7].

Besides the nucleus, trypanosomatids present another DNA compartment that is equally important: the kDNA, which is also called the kinetoplast-DNA network. It is located in one specific portion of a unique single mitochondrion just below the basal bodies, from where the single flagellum emerges. The kDNA is composed of a few maxicircles (20–40 kb) and thousands of minicircles (0.5–10 kb) that are connected like chain mail in medieval armor. The main role of the minicircles is to generate guide RNA, which edits maxicircle transcripts that are required for mRNAs to mature, which in turn encode mitochondrial proteins. Maxicircles contain genes that are responsible for encoding ribosomal RNAs and proteins of the respiratory chain.

Several enzymes distributed in the kinetoplast maintain the kDNA topology. Topoisomerases I and II, DNA polymerase ß and a specific endonuclease (SSE1) are found in the antipodal sites at the network periphery. DNA primase is found in the mitochondrial matrix. In the kinetoflagellar zone, which is a specialized region of the kinetoplast between the kDNA disk and the basal body, there are specific proteins such as universal minicircle sequence binding proteins [8].

The chromatin comprises DNA, histone and nonhistone proteins, in addition to compartmentalized regions and interchromatin spaces. The eukaryotic chromatin contains basic repetitive units called nucleosomes. Each nucleosome consists of a histone octamer (two molecules of the core histones H2A, H2B, H3 and H4), which are associated with a DNA segment that wraps around this octamer twice [3,9,10]. Histones are basic proteins that are rich in lysine and arginine, they are closely associated with DNA, and they are conserved among eukaryotes. The histones of the octamer present two domains: the C-terminal domain, which is found inside the nucleosome, and the N-terminal domain, which is located outside of this structure [9,11].

Another important histone characteristic is that these proteins are subject to post-translational modifications (PTMs), which control gene expression, chromatin assembly, DNA replication, transcription and repair [3,9,10]. About 60 post-translation histone modifications have already been described. Most occur at the N-terminal domain of the protein, but the globular domain has also been described as a site of these modifications. Epigenetic modifications are also related to metabolic functions that act on many biological functions, such as transduction; enzyme activity; the recognition, formation and degradation of proteins; and nucleosome disruption. These modifications also occur as a consequence of the availability of nutrients, hormone stimulation, regulation of the cell cycle and differentiation. Consequently, the progress of PTMs implies successful adaptation and physiology of the cell [12,13].

The reported PTMs in histones include acetylation, deacetylation, methylation, phosphorylation and ubiquitination (Figure 2). Acetylation and deacetylation modulate the affinity of histones for the DNA double helix. This is possible because DNA is negatively charged, whereas the histones are positively charged. This difference allows for a strong association between histones and DNA fragments [14].

**Figure 2.**
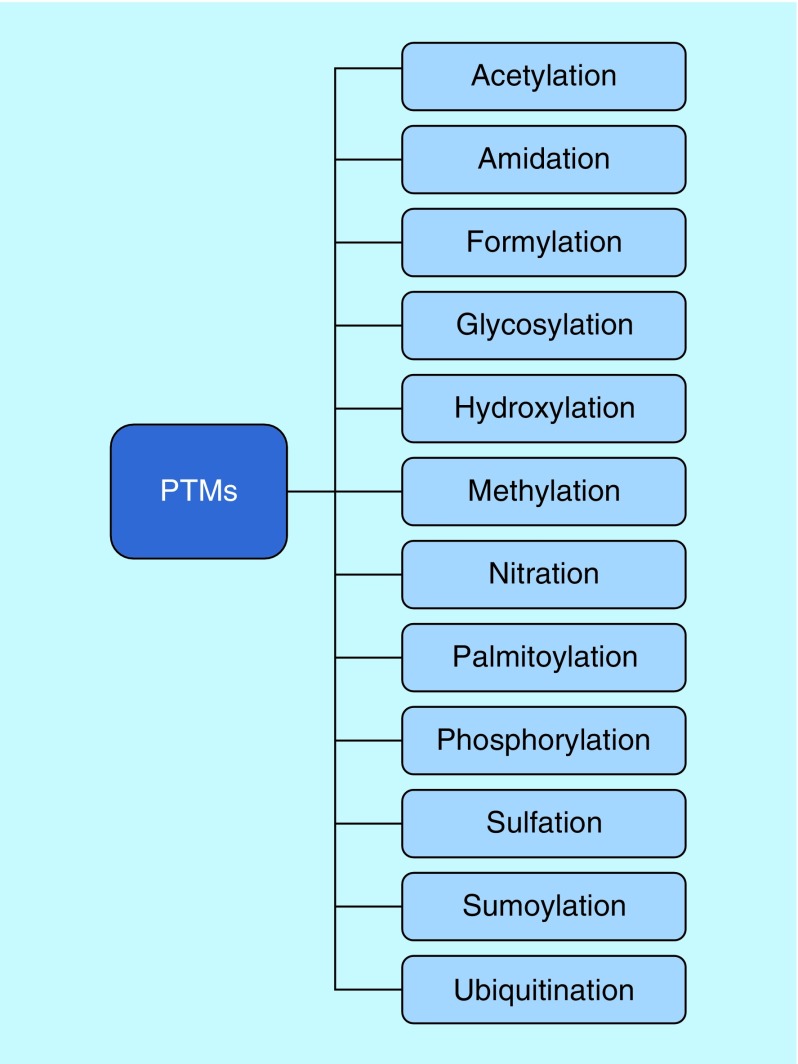
**Post-translational modifications that play a role in several cellular processes.** Acetylation, deacetylation, methylation, phosphorylation and ubiquitination have already been reported in histones.

During acetylation, histone acetyltransferases (HATs) transfer an acetyl group to lysine residues, and the positively charged histones are neutralized. As a result, the octamer disassembles and loses its affinity for DNA, which becomes more relaxed. Thus, the access of transcriptional factors to DNA is facilitated. On the other hand, deacetylation promotes the removal of the acetyl group from lysine residues with the participation of histone deacetylases (HDACs). This leads to a stronger association between histones and DNA, thus increasing its condensation state. Consequently, transcriptional activity is suppressed [14,15], as shown in Figure 3. Histone acetylation is also important for the recruitment of reader enzymes, such as bromodomain proteins, which recognize ε-amino groups and interpret lysine acetylation [16].

**Figure 3.**
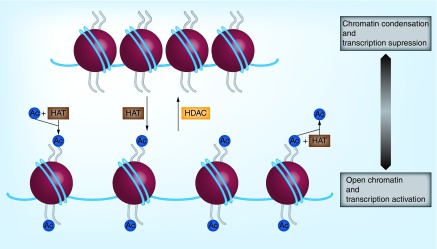
**Histone deacetylation modulates chromatin condensation.** HATs transfer an acetyl group to lysine residues, leading to the neutralization of histones charge. Then, these enzymes lose affinity for DNA and it becomes more relaxed. Consequently, the access to DNA is facilitated. On the other hand, HDACs remove acetyl groups from a lysine amino acid and alter the accessibility of chromatin, which will be more condensed. Inhibition ofhistone deacetylases promotes the increase of histone acetylation and consequently the relaxation of chromatin and transcriptional activation. Ac: Acetyl group.

## Trypanosomatid histones

Histones are basic proteins that are closely related to chromatin. They are comprised of octamers composed of one H3–H4 tetramer and two H2A–H2B dimers. DNA consists of numerous nucleosomes, which are connected to each other by ligand DNA [17]. H1/H5 histones control the dynamics between the presence and absence of this segment of DNA in the nucleosome. Additionally, the H1 histone, as well as other nonhistone proteins, is important for the correct organization and maintenance of chromatin [18].

Histones are also present in protozoa of the Trypanosomatidae family, as well as in higher eukaryotes. These enzymes act on chromatin compaction and undergo PTMs. The annotation of trypanosomatid genomes has enabled the identification of histone genes related to nuclear and mitochondrial DNA, where H1-like histone has been found [11,19].

Previous studies on trypanosomatids histones showed differences between these and human enzymes, such as the N-terminal domain, which exceeds the nucleosome, and the H1 histone, which lacks the globular domain found in other organisms. In addition, *Trypanosoma brucei* presents few H1 histones, and its H3 and H4 have a weak association with the DNA. This suggests that the chromatin condensation does not occur in the same way in trypanosomatids as in higher eukaryotes [20]. Moreover, the structural difference of H1 might explain the nonformation of the highly condensed chromosomes that occur in mammalian cells [21,22]. In addition, separate chromosomes present different clusters where histone genes are clustered in tandem arrays [23].

Trypanosomatids present a single variant of H2A (which is conserved and important for the access of chromatin domains), H2B (a novel trypanosomatid-specific variant) and H3. These variants might be single-copy genes that are incorporated into chromosomes independently of the phase of the cell cycle and replication process [24–26]. Regarding the PTMs of trypanosomatids, histones H4 and H2A are mostly acetylated, whereas H3 and H2B are methylated. Histone H4 PTMs are the best characterized in *T. cruzi*. The H2A histones can also be hyper-acetylated at the C-terminal domain, which could facilitate the access of RNA polymerases to the chromatin [27–29].

In addition, the lysine residues that are acetylated vary between species, and the distribution of acetylated histones is different inside the nucleus. For example, *T. cruzi* H4 is preferentially acetylated at Lys57, whereas in *T. brucei*, H4 is preferentially acetylated at Lys2 and Lys5. H4 acetylated at lysine 4 (H4-K4Ac) is found next to the nuclear envelope and in chromatin-rich areas. H4-K10Ac and H4-K14Ac are present in more discrete domains inside the nucleus [30].

Histone synthesis occurs during the S phase of the cell cycle. In trypanosomatids, the control of histone gene transcription is a post-translational event, whereas in eukaryotes, the gene expression generally takes place during transcription [31]. The polyadenylation mechanism is also distinct between Trypanosomatidae protozoa and other eukaryote organisms. In eukaryotes, histone mRNAs are not polyadenylated and interact with stem-loop binding protein, which favors the mRNA half-life during S phase. However, trypanosomatid histone mRNAs are polyadenylated, and these protozoa also present stem-loop binding protein similar to protein sequences in their genome [31–33].

**Figure 4.**
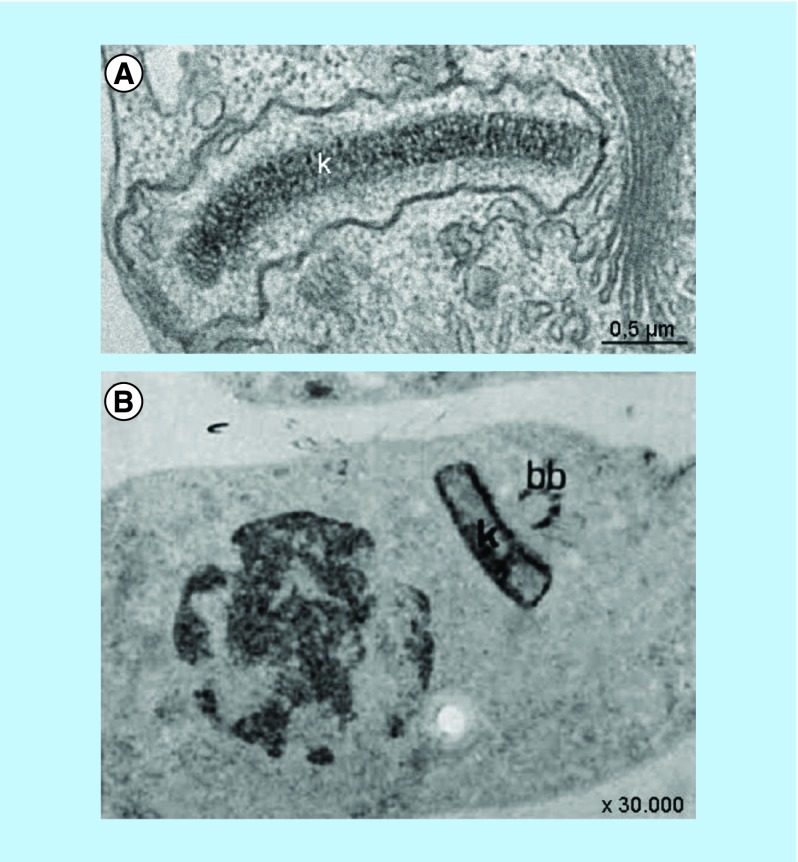
**Mitochondrial DNA organization as observed by transmission electron microscopy.** **(A)** Mitochondrial DNA topology of epimastigote form of *T. cruzi*. Note the characteristic electron density through the disk. **(B)** Ethanolic phosphotungstic acid method, showing the more intense staining of the periphery of the disk, indicating the presence of basic proteins. Note that the nucleus of *T. cruzi* is stained as well, which shows the distribution of histones [34].

In general, the discussion of DNA organization refers to nuclear DNA and histones. However, in the case of trypanosomatids, it is fundamental to consider the mitochondrial-kinetoplast DNA. Early cytochemical studies showed the presence of basic proteins in the kinetoplast, which was revealed by labeling of the structure using silver ammoniacal particles and ethanolic-phosphotungstic acid technique [34,35], as shown in Figure 4. The mitochondrial DNA of trypanosomatids is highly organized and, in some cases, very compact. Several enzymes that play a role in kDNA replication and even in its topology are present on opposite sides of the kinetoplast disk (called antipodal sites), in the kinetoflagellar zone, or throughout the kDNA fibers.

One class of these enzymes is the kinetoplast associated proteins (KAPs). The characterization of *Crithidia fasciculata* KAPs has demonstrated that these enzymes are small, basic, have similar composition when compared with H1 histone, and have specific localization at the kinetoplast [33]. Regarding their functions, KAPs have been related to the interaction between minicircles and to kDNA organization in *C. fasciculata* [34]. This can be observed after the knockout of *CfKAP1*, which leads to changes in the kDNA network [36–38].

In *T. cruzi*, KAP3, KAP4 and KAP6 have been characterized as basic proteins and shown to play a role in kDNA arrangement [38]. The knockout of *TcKAP3* did not impair parasite proliferation, differentiation or infectivity. It is possible that the absence of this KAP may be compensated by other KAPs, such as KAP4 or KAP6, which help to maintain kDNA topology during different evolutive forms of this protozoan [38].

## Histone deacetylases

HDACs are divided into two major groups: the Zn^2+^-dependent HDACs and the NAD^+^-dependent sirtuins (SIRT). The Zn^2+^-dependent HDACs include classes I, II and IV. Class I includes HDACs 1, 2, 3 and 8, which are found in the nucleus. Class II comprises class IIa (including HDACs 4, 5, 7 and 9) and class IIb (which includes HDACs 6 and 10). In contrast to class I, class II enzymes are present in the cytoplasm and are able to shuttle in and out of the nucleus. Class IV is more related to class I and comprises HDAC 11 [14].

SIRT includes class III and is known to have nonhistone proteins as a substrate. These enzymes are able to remove acetyl residues, consuming nicotinamide adeninedinucleotide (NAD^+^) and generating nicotinamide, O-acetyl-ADP-ribose and the deacetylated substrate [39]. In general, histone and nonhistone proteins are substrates for HDACs. Among their substrates are DNA-binding proteins, transcriptional regulators and cytoskeleton proteins. Thus, HDACs play a role in diverse cellular processes, from the control of gene expression to cell death mechanisms [40–45].

One approach to investigating how these enzymes act is using knockout models. For example, HDAC1 is related to cell proliferation and gene expression during mouse embrio development, and its knockout leads to embryonic lethality. In the case of HDACs 2, 5 and 9, knockout mice present cardiac defects. Regarding HDAC4, the occurrence of defective chondrocyte differentiation has been reported, and there were vascular defects for HDAC7 [44,46]. HDAC6 is a cytoplasmic class II enzyme that deacetylases nonhistone proteins. One of these proteins is α-tubulin, which is present in the microtubules that compose the cytoskeleton. Thus, HDAC6 plays a role in the organization and dynamics of the cytoskeleton [47].

The alternation between the higher and the lower compaction of DNA by HATs and HDACs controls the suppression and activation of genes. When HATs are inactive or HDACs are overexpressed, tumor cell proliferation can be facilitated [48]. For example, higher levels of HDAC 2 and 3 have been found in cases of colon cancer. HDAC1 has been observed in gastric and breast cancer, while HDAC 5 and 10 have been observed in lung cancer. Moreover, cancer is not just related to the balance of acetylation and deacetylation, but to specific histone modifications. In 2005, Fraga *et al*. showed that a typical characteristic of tumor cells is when the acetylation of histone H4 at lysine 16 and the trimethylation of histone H4 at lysine 20 are lost [49]. The inhibition of HDAC promotes cell cycle arrest by the downregulation of cyclins A and D; the activation of the death-receptor and intrinsic apoptotic pathway, leading to cell death; and the activation of the immune response [50,51].

## Trypanosomatid HDACs

Several class I, II and III HDACs have already been found in trypanosomatids, but not all enzymes present in mammalian cells are found in these parasites. According to the TriTryp gene bank, trypanosomatids have a gene encoding three Sir2-related proteins (SIR2RPs) [52].

*Trypanosoma brucei* presents four HDAC orthologs of classes I and II: *TbDAC1*, *TbDAC2*, *TbDAC3* and *TbDAC4*. Regarding sequence similarity, *TbDAC1* and *TbDAC2* are more related to human class-I HDACs, whereas *TbDAC3* and *TbDAC4* to class II HDACs. Concerning the role and localization of these enzymes, TbDAC1 and TbDAC3 are localized in the nucleus, participate in the silencing of VSG genes and are essential for *T. brucei* survival. TbDAC2 and TbDAC4 are found at the cytoplasm and the latter is required for cell cycle progression at G2/M phase.

Three sirtuin homologs are present in *T. brucei*: *TcSIR2RP1*, *TcSIR2RP2* and *TcSIR2RP3*. The first one is found in the nucleus and known for its role on DNA repair, VSG expression and parasite survival, occurring in all developmental stages of the parasite [53–58]. In contrast, TbSIR2RP2 and TbSIR2RP3 are found in the mitochondrion, but are not essential for parasite proliferation and differentiation. Curiously, SIR2RP2 is not present in *T. cruzi*. However, this protozoan has TcSIR2RP1 and TcSIR2RP3, which are localized in the cytoplasm and mitochondrion, respectively. The same study that reported the localization of these proteins analyzed the consequences of their overexpression. Epimastigote proliferation was not affected after TcSIR2RP1 overexpression but it was inhibited when TcSIR2RP3 was overexpressed. On the other hand, the rate of metacyclogenesis was increased with the overexpression of TcSIR2RP1, but did not change after TcSIR2RP3 overexpression [59].

*Trypanosoma cruzi* has two coding sequences for lysine deacetylases that are homologous to HDAC6 and another two for sirtuins (SIR) (http://tritrypdb.org/tritrypdb/). TcSIR2RP1 is localized in the cytosol and TcSIR2RP3 in the mitochondrion, and their superexpression is related to *T. cruzi* growth and differentiation blockade, which evidences the importance of these enzymes to parasite life cycle completion [58].

In *Leishmania*, there are four genes for HDACs of classes I and II, and three for sirtuins. A relationship has been reported between the differentiation of promastigote to amastigote and the upregulation of HDAC. The presence of a class III HDAC homologue (*LmSIR2RP1*) on promastigote and amastigote cytoplasm has been described in cytoplasmic granules. Moreover, the overexpression and gene disruption of SIR2RP1 suggest that this protein is required for protozoan survival [29,60–62]. The protein SIR2RP1 present in *L. infantum* can deacetylase α-tubulin and is thus correlated to cytoskeleton organization. It is also important to mention that SIRT2 and HDAC6 act similarly in humans [56,57].

## HDAC inhibitors

HDAC inhibitors (HDACi) are compounds that target HDACs and prevent the removal of acetyl radicals, which interferes with condensation of chromatin condensed or affect transcriptional factors by increasing their acetylation levels [63]. In general, HDACi lead to the upregulation of pro-apoptotic and downregulation of anti-apoptotic proteins, resulting in apoptosis; oxidative damage to DNA; the promotion of mitosis delay; the inhibition of hsp90, which is necessary for intracellular signaling; and the activation of autophagy [64,65]. HDACi have also been successfully used in combination with distinct enzymes inhibitors such as proteasome and DNA methyltransferase inhibitors in order to improve the effectiveness compared with the drug alone [66–68]. Furthermore, the use of HDACs and HAT inhibitors has provided promising results that stimulate the development of new compounds by medicinal chemistry aimed at epigenetic modifications [69].

There are natural and synthetic compounds known for inhibiting the different classes of HDACs, which are divided into hydroxamic acid derivatives, cyclic peptides, short-chain aliphatic acids and benzamides [43,44,70]. Table 1 presents the chemical structure of some of these compounds. The natural compound trichostatin A (TSA) was the first HDAC hydroxamate inhibitor to be discovered. This compound was isolated from *Streptomyces hygroscopicus* and was known for its antibacterial properties at the time [71]. Currently, TSA is known for inhibiting HDAC6. At nanomolar concentrations, it can inhibit cell proliferation by histone hyperacetylation [10,64].

**Table 1.** **Structure and classification of some histone deacetylase inhibitors.**

**Class**	**Compound**	**Structure**
Hydroxamates	Trichostin A	

	Vorinostat (SAHA)	

	Belinostat	

	Panobinostat	

	Tubastatin A	

Nicotinic acid derivative	Nicotinamide	

Indole derivative	KH-TFMDI	

Reverse amide	Salermide	

Cyclic peptide	Romidepsin	

Significant effects of TSA on *Plasmodium falciparum* growth have been described [72]. However, despite its effectiveness, its high toxicity discourages its continued use in chemotherapeutic studies [8,64]. TSA has also been evaluated against *Toxoplasma gondii* and *T. brucei*, presenting low activity for *T. gondii* and inhibiting cell growth for *T. brucei* [73,74]. Campo [75] evaluated the effects of TSA (a class I/II HDACi) and sirtinol (a class III HDACi) on *T. cruzi*. Although TSA and sirtinol did not inhibit epimastigote proliferation with 40 and 100 μM, they were able to block metacyclogenesis. In *T. cruzi*, the effects of different HDACi may also be life-stage specific, as demonstrated by Campo [75]. This comparative study showed that in the presence of the trypomastigote forms of these drugs was more infective, whereas the differentiation from epimastigote to the infective form was blocked. These data suggest that the inhibition of different classes of HDACs interferes with the gene expression and metacyclogenesis of parasites.

Another generation of compounds comprises hydroxamic derivatives, such as SAHA (also known as Zolinza and vorinostat). SAHA acts on classes I and II (HDAC1, 3 and 4) and can down-regulate components of the DNA replication complex (such as MCM complex) and DNA repair genes (such *Rad51, Rad54* and *BRCA2*). This drug is effective at nanomolar concentrations and was approved by the US FDA in 2006 for clinical use against cutaneous T-cell lymphoma [3,76,77]. Against *T. gondii* SAHA presented better selectivity than TSA [78].

The third group of HDACi includes the short-chain fatty acids butyrate, phenylbutyrate, valproate and phenylacetate. Valproic acid and phenylbutyrate have not yet been characterized as HDAC inhibitors, but, due to their low cytotoxicity, they have already been used in chemotherapy. Like TSA, the short-chain fatty acid butyrate promotes histone hyperacetylation and inhibits tumor cell proliferation [42,44].

The well-known anticancer drugs SAHA, belinostat, panobinostat and romidepsin have already been used against pathogenic parasites *Plasmodium knowlesi*, *Schistosoma mansoni*, *Leishmania amazonensis* and *L. donovani*. *P. knowlesi* was more susceptible to treatment than the other species. *Leishmania* amastigotes and promastigotes were resistant when the concentrations used were lower than 20 μM. This effect was similar to that on *S. mansoni* schistosomula, except for the use of romidepsin, which was able to inhibit parasite egg production [79].

Another study demonstrated the potential of HDACs as chemotherapeutic targets against pathogenic parasites by the evaluation of 1.200 approved drugs against *Cryptosporidium parvum*. In this case, SAHA presented anticryptosporidial activity *in vivo* and *in vitro*, and inhibited sporozoites HDACs (IC_50_ = 90 nM) [80].

In 2002, Kelly *et al*. evaluated the effect of several hydroxamic acid derivatives that are known to inhibit human HDACs on the cultured bloodstream form of *T. brucei*. The growth of this form of *T. brucei* was impaired by three classes of hydroxamic acid derivatives at nanomolar concentrations, such as an IC_50_ of 34 nM. Moreover, the class of sulphonepiperazines compounds caused parasite death after only 4 h of treatment with micromolar concentrations [81].

Another study evaluated the potential of two known anticancer HDACi (panobinostat and belinostat) under the following conditions: separately, and in combination with pentamidine, suramin, melarsoprol or nifurtimox. The aim was to inhibit *T. brucei brucei* proliferation at concentrations that are not toxic to patients. However, parasite growth was not affected by this condition and there were no successful synergistic combinations. Further analysis suggested that trypanosome HDACs present some specificity because no correlations between the effects against human HDAC isoform and parasite proliferation were found [82].

Engel *et al*. [83] used belinostat and panobinostat as well as SAHA and romidepsin (all FDA approved drugs) against *P. falciparum* and *T. brucei brucei*. These HDACi were not more effective against African trypanosomes than mammalian cells. On the other hand, these compounds were more selective for *P. falciparum* and inhibited the growth of the asexual stage.

**Figure 5.**
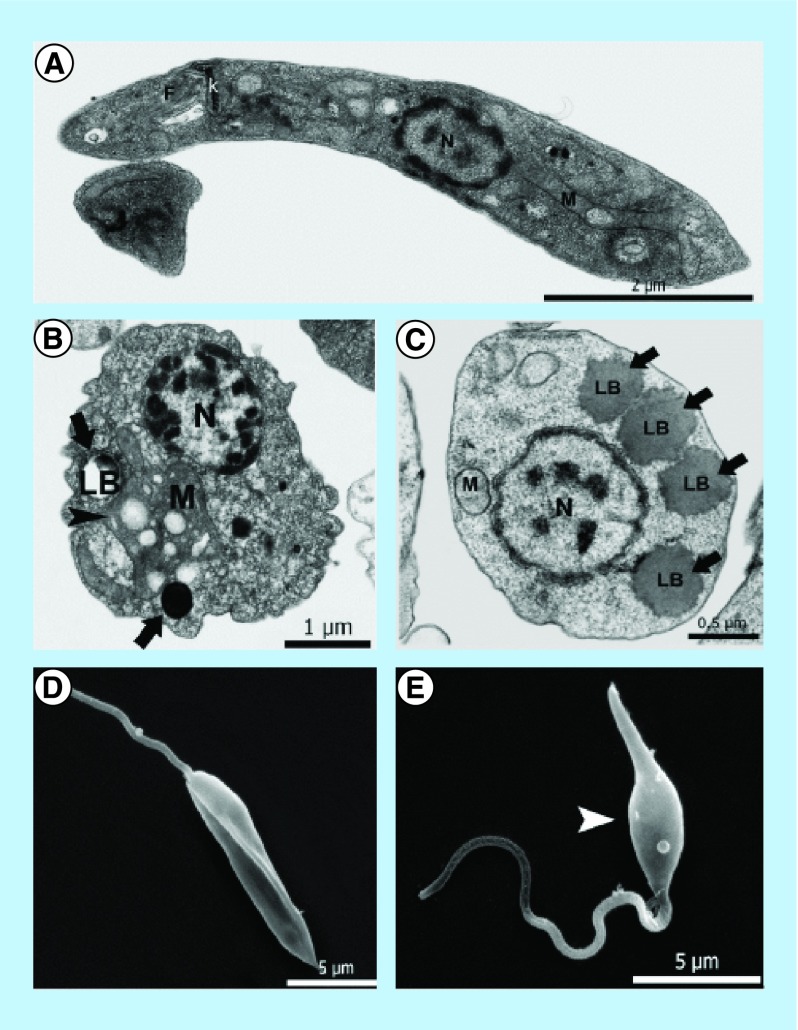
**Analysis of the effects of KH-TFMDI on *Leishmania amazonensis* ultrastructure.** **(A)** Transmission electron microscopy of nontreated *L. amazonensis*, showing typical structures of the family, such as kDNA (k), flagellum (f), nucleus (n) and mitochondrion (m). **(B)** Parasite treated with 10 μM KH-TFMDI for 48 h. The arrows show lipid bodies, and the arrowhead shows mitochondrial alterations. **(C)** *L. amazonensis* with 5 μM KH-TFMDI for 48 h. Note the presence of several lipid bodies (LB) in the cytoplasm. **(D)** Nontreated *L. amazonensis* by scanning electron microscopy. Note the elongation of the cell body. **(E)** Treatment with 3 μM KH-TFMDI for 72 h. Parasites were swollen in the central region of the body (arrowhead). Reproduced with permission from [85].

A novel 3-arylideneindolin-2-one (KH-TFMDI), a sirtuin (class III HDAC) inhibitor, was evaluated against *T. cruzi* with concentrations ranging from 0.5 μM to 2.5 μM. Under these conditions, this compound promoted the inhibition of amastigote replication, bloodstream trypomastigote lysis, epimastigote cytokinesis impairment and ultrastructural modifications in the Golgi complex and nuclear envelope. It was also more effective than the reference drug benznidazole [84]. The effects of this compound have also been tested against *L. amazonensis*, presenting IC_50_ at very low micromolar concentrations. The treated parasites presented cytoskeleton disorganization, increased expression of acetylated tubulin, and atypical chromatin condensation [85], as shown in Figure 5.

Sir2 inhibitors were subjected to a virtual screening, which revealed that nicotinamide is the best ligand. Nicotinamide is a well-known sirtuin inhibitor and a water-soluble vitamin of the B complex. It caused alterations in the mitochondrion, kinetoplast and vacuolization in the cytoplasm of epimastigotes of *T. cruzi*. Furthermore, it reduced the number of amastigotes in infected macrophages and promoted lysis in trypomastigotes [86,87].

Salermide is a novel derivative of sirtinol and has been tested against *T. cruzi*. Its effects have also been compared with nicotinamide. Treatment for 72 h with 5 μM of salermide inhibited the proliferation by 50% and reduced the cell viability of epimastigotes. On the other hand, in the presence of nicotinamide, the same result was obtained with concentrations higher than 25 mM. Salermide was also effective against infective forms of *T. cruzi* by reducing the parasitemia of infected BALB/c mice by approximately 40% after treatment [88].

Tubastatin A is also an HDAC6 inhibitor such as TSA. However, this compound stands out because it is specific and the most effective HDAC6 inhibitor evaluated thus far [89]. Tubastatin A can inhibit tumor cell proliferation [90]. At nanomolar concentrations, Tubastatin A inhibited the proliferation of melanoma cells and caused cell cycle arrest in the G1 phase [91]. The treatment of *L. donovani* with Tubastatin A showed that this compound is more cytotoxic to promastigotes (IC_50_ of 4.4 μg/ml) than amastigotes (IC_50_ of 8.8 μg/ml). This points to the importance of HDAC6 for the viability of the intracellular forms and the different effects of the same drug on each evolutive form of the parasite, as similarly reported for *T. cruzi* [92]. Table 2 shows a summary of IC_50_, CC_50_ and LC_50_ values of different compounds against trypanosomatids and table two shows the structure and classification of some HDACi.

**Table 2.** **IC_50_^1^, LC_50_^2^ and EC_50_^3^ values of different compounds against trypanosomatids.**

**Inhibitor**	**Parasite**	**IC_50_**	**LC_50_**	**EC_50_**
Trichostatin A	*T. brucei* (bloodstream)	–	–	6363 (4144–9770) μM – 48 h

Sulphonepiperazine analogue	*T. brucei* (bloodstream)	34 nM	–	1577 (1325–1878) μM – 48 h

Panobinostat	*T. brucei* (bloodstream)	–	–	2156 (1747–2662) μM – 48 h

Belinostat	*T. brucei* (bloodstream)	–	–	2751 (1917–3947) μM – 48 h

Tubastatin A	*T. brucei* (bloodstream)	–	–	–

	*L. donovani* (promastigote)	4.4 μg/ml – 72 h	–	–

	*L. donovani* (amastigote)	8.8 μg/ml – 72 h	–	–

	*T. cruzi* (amastigote)	0.5 (+/-)0.2 μM	–	–

	*T. cruzi* (epimastigote)	7 (+/-)0.51 μM	–	–

	*T. cruzi* (trypomastigote) 4°C	–	0.8 (+/-) 0.3 μM – 24 h	–

	*T. cruzi* (trypomastigote) 37°C	–	2.5 (+/-)1.1 μM – 24 h	–

KH-TFMDI	*L. amazonesis* (amastigote)	4735 μM – 24 h 2341 μM – 48 h 1976 μM – 72 h	–	–

	*L. amazonesis* (promastigote)	2367 μM – 24 h 2438 μM – 48 h 1976 μM – 72 h	–	–

	*T. cruzi* (epimastigote) Y strain	0.037 (+/- +0.016 mM – 72 h 0.126 (+/-) 0.025 mM 144 h	–	–

Nicotinamide	*T. cruzi* (epimastigote) Columbian strain	0.025 (+/- 0.001 mM – 72 h	–	–

	*L. infantum* (promastigote)	13.9 mM (+/-) 4.2 mM	–	–

	*L. infantum* (amastigote)	5.5 mM (+/-) 0.5 mM	–	–

Salermide	*T. cruzi* (amastigote)	2 μM – 24 h and 48 h	–	–

	*L. donovani* (promastigote)	25.7 μg/ml – 72 h	–	–

SAHA	*L. donovani* (amastigote)	> 40 μg/ml – 72 h	–	–

Information taken from [74,78,81–88,92,93].

EC_50_: 50% cytotoxic concentration; IC_50_: Half maximal inhibitory concentration; LC_50_: Lethal concentration required to kill 50% of the population.

Heimburg *et al*. [94] screened several novel HDAC inhibitors that target *S. manosni* HDAC8, which is expressed during all life stages of this parasite and that therefore it becomes a potential target. At 10 μM and 20 μM after up to 2 days of treatment, the compound called 13l was lethal to schistosomula and promoted the separation of 90% of male and female adults after 5 days of treatment.

Although inhibitors currently tested in protozoa are not selective for these organisms, the results described in the literature reinforce the idea that these enzymes are susceptible to inhibition, which leads to proliferation reduction and also cell death, among other effects. The differences between human and trypanosomatids HDACs are of extreme importance so that they can be used selectively as a therapeutic target. Moreover, the interface between biology and the advances in the area of medicinal chemistry can favor the modification of known molecules, which could target the enzymes of the parasite, and not of the host.

The treatment with HDACi has been related to changes in cell cycle progression. One of the reasons is the induction of the p21 protein-encoding gene. This cyclin-dependent kinase regulates the transition from G1 to S, and the respective genes are some of the most commonly activated genes in the presence of HDACi. Consequently, the presence of HDACi in cell cultures blocks the cell cycle in the G1 phase. Furthermore, the cell cycle may be arrested in G2, preventing mitosis. This results from the high levels of acetylation in regulatory proteins that play a role during the S/G2 progression [63]. The increase of histone acetylation levels induced by HDACi also contributes to an inefficient cell cycle, since its consequences are a loss of function of spindle checkpoint proteins and heterochromatin binding proteins [95,96].

The capability of HDACi to arrest the cell cycle is also due to the downregulation of cyclins and other proteins involved in the progression and control of the cell cycle. Moreover, the concentration of the drug used during treatment can influence the phase of the cell cycle that is arrested. For example, G1 arrest is induced in the presence of low concentrations of HDACi, and cell cycle blockade is induced at G1 and G2/M with high concentrations. This effect may be caused by the increase of cyclin-dependent kinase inhibitors and the decrease of cyclins [42,97].

It is still not clear what the mechanisms of action of HDACi are or what the specific HDAC target of each inhibitor is. However, their uses in chemotherapy include combination with a variety of compounds, such as anthracyclines, topoisomerases inhibitors, antiangiogenic agents, proteasome inhibitors and apoptosis inducers [98,99]. Furthermore, there is evidence that combinations of HDAC with other classes of inhibitors are more effective than HDACi monotherapy [100]. Another important aspect that must be considered is the ability of HDACs to form multiple regulatory complexes. Knowledge about these complexes might contribute to the development of compounds that can inhibit a specific complex and not just a single HDAC, culminating in more effective therapies [68].

The drug resistance problem is related to a set of factors: the low availability of drugs so far, the complexity of the protocols for drugs administration, and by genetic and molecular characteristics of the parasites, such as the capacity of modifying their target molecule, involving mutations causing loss of the uptake system, changing in the function or quantity of surface transporters, inactivation of drugs, excretion and relocation into vacuoles [101].

Regarding Chagas disease, the severe side effects caused by benznidazole are one of the factors responsible for treatment failure, which might be mistaken for treatment discontinuity or for drug resistant parasites [102].

To achieve a successful treatment, it is important to take into account the possible prior exposure to the pathogen, the genetic background of the patient and to understand the mechanisms that cause resistance in parasites [103,104]. Some genes responsible for drug resistance have already been identified and their detailing can contribute towards progress against treatment failure [102]. Moreover, compounds that are able to inhibit ABC transporters and drug delivery systems (such as nanoparticles, which increases efficacy) have been used as an alternative to reverse resistance [105].

## Conclusion

Trypanosomatid parasites are the causative agents of leishmaniasis, Chagas disease, and sleeping sickness, thus being a public health problem throughout the world. Many years after their discoveries, their treatments have produced resistant parasites. The development of new drugs and the identification of new molecular targets are crucial to treatment success. In this sense, the existing knowledge about HDACs, including their role and importance for parasite survival, can be exploited to fill this gap.

## Future perspective

The importance of epigenetic regulation for cellular metabolism has created interest in the design of drugs that target enzymes involved in the acetylation of DNA and the PTM of histones. Epigenetic regulation, mediated by the condensation level of chromatin, is important for gene activation or inactivation, enzyme activity, antigenic variation, virulence and also differentiation in protozoa. Thus, epigenetics are involved with the establishment of parasite infection. The importance of epigenetic regulation for cellular metabolism has created interest in the design of drugs that target enzymes involved in the acetylation of DNA and the PTM of histones. The various reports collected in this review demonstrate that the enzymes involved with the epigenetic processes, such as HDACs, are promising targets because of the many effects that their inhibition promotes. However, there is a lack of molecules with specificity for the catalytic site of HDACs and limited knowledge about their mechanism of action in signaling pathways. Nevertheless, it is necessary to investigate the effects of different drugs against HDACs and relate them with their chemical structures to enhance the potential of these compounds as chemotherapeutic agents, besides improve their pharmacokinetic properties.

The combination of drugs has been used with distinct classes of compounds, including HDACi, as an alternative to minimize toxicity, reduce side effects and enhance treatment efficiency. In literature, there are reports about the association between HDACi and drugs that act on DNA repair pathways, proteasome inhibitors and topoisomerases. In the latter case, which includes studies with preclinical trials, the association promoted higher accumulation of the topoisomerase inhibitor in the nucleus, DNA damage and cell death [106]. However, it is still necessary to make HDAC inhibitors more specific for their targets and in the smallest concentrations possible. Regarding antiparasitic therapy, one approach is to evaluate the effects of known antitumor drugs against pathogenic parasites. Furthermore, the applicability of new compounds to the antiparasitic chemotherapy should take into account the selectivity of the compound for pathogenic parasites (including the resistant ones), as well as low treatment cost, and combination therapy with other potential compounds.

**Executive summary**

- Post-translational modifications control gene expression, chromatin assembly and DNA replication, transcription and repair.
- Trypanosomatid histones are different from those of humans, which make them interesting targets.
- Histone deacetylases are involved in distinct cellular processes, such as gene expression control and cell death mechanisms.
- Epigenetic regulation has been explored in chemotherapeutic studies and for drug design as a promising alternative, including against pathogenic parasites.
